# Valorization of textile waste for removal of Cadmium from contaminated water

**DOI:** 10.1038/s41598-024-82456-x

**Published:** 2024-12-23

**Authors:** Humera Aziz, Munir Ashraf, Muhammad Rizwan, Umair Riaz, Saba Akram, Ali Raza, Jean Wan Hong Yong

**Affiliations:** 1 Department of Environmental Sciences, Government College University, Faisalabad, 38040 Pakistan; 2 School of Culture and Design, Clothing Technology, HTW Berlin –University of Applied Sciences for Technology and Economics Berlin, Berlin, Germany; 3 Department of Soil and Environmental Sciences, MNS-University of Agriculture, Multan, 60000 Pakistan; 4 Functional Textiles Research Group, School of Engineering and Technology, National Textile University, Faisalabad, 38000 Pakistan; 5 Faculty of Veterinary Sciences, University of Veterinary and Animal Sciences, Lahore, 54000 Pakistan; 6 Department of Biosystems and Technology, Swedish University of Agricultural Sciences, Alnarp, 23456 Sweden

**Keywords:** Recycling, Post-consumer textile waste, Functionalization, Carboxylic acid groups, Sorption, Wastewater treatment, Sustainability, Environmental sciences, Natural hazards

## Abstract

The rapid development in agriculture and industrial sectors has raised some serious global issues like heavy metals pollution of water resources. Cadmium (Cd) is amongst the major water pollutants worldwide. In this study, two novel sorbents were prepared by using post-consumer textile waste (PCTW). The waste denim fabric was recycled with environmentally friendly H_2_O_2_ and Ozone through oxidation under alkaline conditions to produce several functional groups at the surface (named as ODF@H_2_0_2_ and ODF@0_3_ respectively) that could trap the heavy metal cations from contaminated water. The functionalized fabric sorbents were characterized by Fourier transform infrared spectroscopy (FT-IR), X-Ray diffraction (XRD), scanning electron microscopy (SEM), and energy dispersive X-Ray (EDX) that revealed the presence of carboxylic acid, hydroxyl, and amine functional groups on their surfaces. The sorption isotherm, sorption kinetics and sorption thermodynamics were carried out to unravel the sorption process mechanism. The ODF@H_2_0_2_ sorbent was proved more effective by giving maximum adsorption capacity of (238.09 mg g^−1^) compared to Ozone treatment (175.44 mg g^−1^) for Cd^2+^ and achieved within just 20 min for both sorbents. Pseudo 2nd order and Langmuir models confirmed the chemosorption as dominant mechanism for the monolayer sorption of Cd^2+^ ions onto ODF@H202 and ODF@0_3_. The sorption thermodynamic revealed the sorption process as endothermic and spontaneous in nature. The results showed that both ODF@H_2_0_2_ and ODF@0_3_ sorbents have an efficient potential for sorbing Cd from contaminated water. Furthermore, both ODF@H_2_0_2_ and ODF@0_3_ sorbents were also tested in a regeneration study to investigate the reuse of these sorbents, and we achieved marvelous results. Both sorbents gave up to 90% of the sorption capacity even after 10 recycles. Conclusively, both sorbents can have their implications for the preparation of filters that can be used for the treatment of wastewater. This study has practical significance by tackling the two environmental problems i.e. heavy metal pollution and denim waste.

**Supplementary Information:**

The online version contains supplementary material available at 10.1038/s41598-024-82456-x.

## Introduction

Though this is the era of growing technology, still the concentration of heavy metals in water is not within the safe range in many countries as suggested by regulatory authorities^1^. Mostly, the water is contaminated with various heavy metals like cadmium, arsenic, mercury, lead, and chromium that are hazardous to human health^2^. These heavy metals pollution of water and soil has become the hotspot of recent studies for environmental researchers due to their non-biodegradable, bioavailable, persistent, and more toxic nature^3^. Due to increased consumption of these heavy metals through drinking of contaminated water, diseases like diabetes, neuronal damage, cardiovascular disorders, renal injuries, and even cancer have been diagnosed^3,4^. Cd has been listed as the fifth harmful toxicant for human beings which causes diseases like osteoporosis, hypertension, diabetic renal dysfunction/ complications, pancreas, bladder, kidney, lung and breast cancer^5,6^. The main reason behind the heavy metal toxicity is the generation of reactive oxygen species that are responsible for oxidative damage and health associated disastrous effects^7^. The mortality rate is also increasing due to excessive utilization of various heavy metals^8^.

Another issue of same importance is the post-consumer textile waste (PCTW) due to fast fashion. It is reported that 27 million tons of cotton are being produced per year globally and almost the same number of textiles in the form of PCWT are disposed of every year. About 5% of all worldwide landfills are being taken up by dumping of textile waste which is creating serious environmental and health hazards due to open air dumping^9,10^. The degradation of textile waste particularly cotton generates greenhouse methane (CH_4_) gas and carbon dioxide (CO_2_) which are responsible for climate change while, toxic chemicals and dyes have been leached out into soil and groundwater causing soil and freshwater pollution.

Recently, every organization has been trying to achieve sustainability by recycling waste and converting it into useful products that can have minimum environmental impacts^11^. The denim, which is generally made of cotton, loses its aesthetic and functional value at the end of its lifecycle^11–13^. Moreover, recycling of post-consumer denim waste into new textile products is also very challenging due to loss of fiber strength and length. Denim fabric is often made from cotton having more than 90% cellulose. Cellulose is made up of several functional groups like hydroxyl, carbonyl and others. Due to the presence of hydroxyl groups on its surface the cellulose can be converted into sorbents having potential to extract positive metal ions from a contaminated medium^14^. These functional groups can further be oxidized to convert hydroxyl ion into carboxylic acid groups having more strong potential to sorb heavy metal ions compared to hydroxyl groups^14–16^.

To address the issues of heavy metals in water and denim waste environmental footprint, the present study is aimed at recycling denim waste and preparing a sustainable and cost-effective sorbent using environmentally safe hydrogen peroxide and ozone for generation of carboxylic acid groups on denim waste. Moreover, the present study also explores the possibility to regenerate sorbent once it is saturated with heavy metals during removal process. This study has a practical significance as it will tackle two environmental problems at the same time. This study is novel as it involves preparing two sustainable sorbents by recycling waste material into useful and reuseable sorbents having strong sorption potential for metal ions. Both sorbents are environmentally friendly as no harmful chemicals are used during their synthesis and will have minimum environmental impacts.

## Results and discussion

### Selection of efficient oxidation treatment

**Fig. 1:**
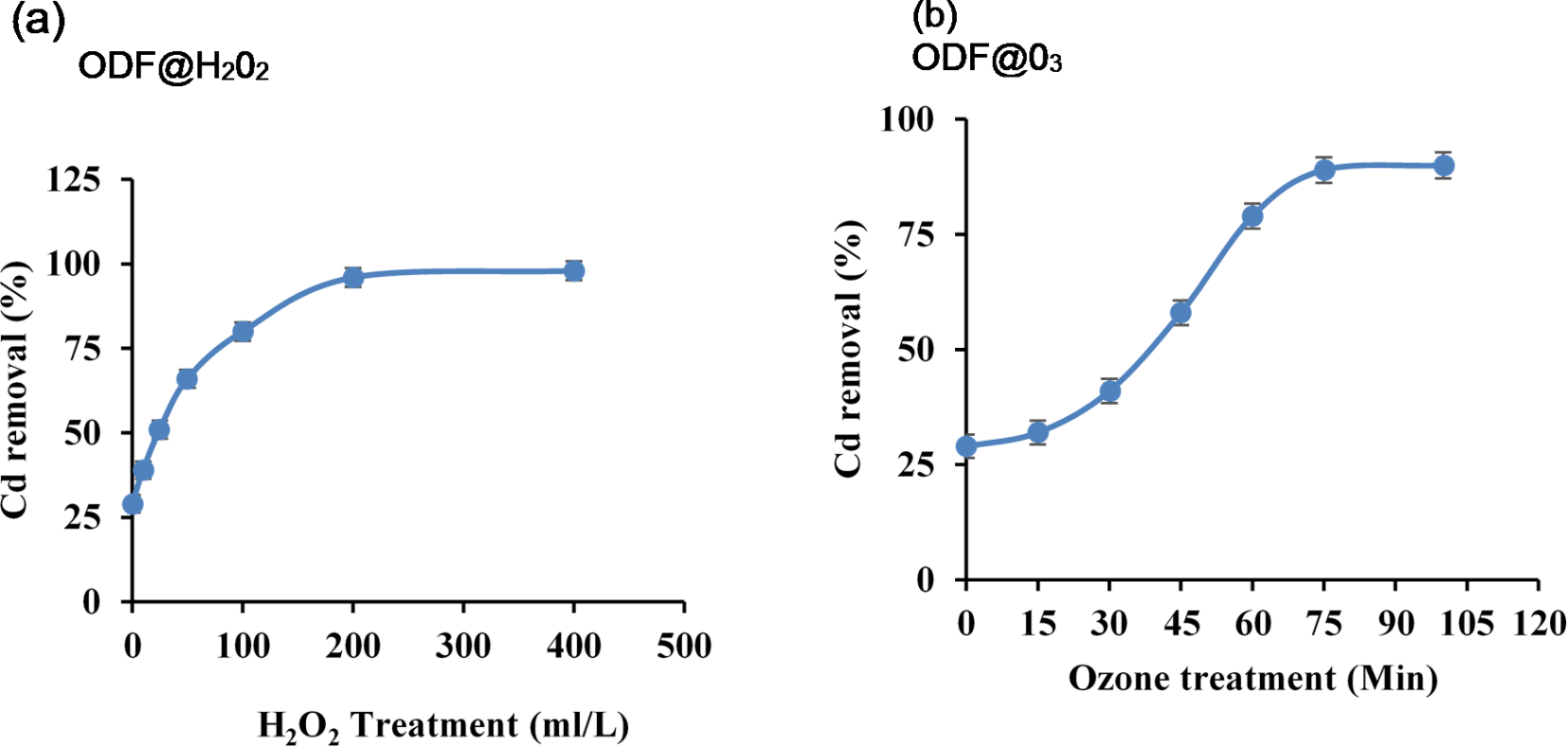
Cadmium removal percentage achieved with different levels of H_2_O_2_ (**a**) and Ozone treatments (**b**).

The effect of different oxidation treatments on the removal of Cd was studied by varying the oxidation treatments for H_2_O_2_ (0, 10, 25, 50, 100, 200, 400 ml/L), and for Ozone (0, 15, 30, 45, 60, 75 min). The adsorbent dose (0.01 g), shaking time (1 h) and Cd concentration (100 ppm) were kept constant and different H_2_O_2_ and ozone treatments were tested as described above. These samples were run on Atomic Absorption Spectrophotometer (AAS) and Cd removal percentage was calculated (Fig. 1a and b). We achieved maximum Cd removal percentage (96%) with H_2_0_2_ treatment 200 ml/L (ODF@H_2_0_2_) and 89% with Ozone treatment of 75 min (ODF@0_3_) compared to fabric with untreated denim fabric (UDF) which gave only 29% Cd removal percentage. Both above samples were chosen for further use in the experiment.

## Analysis of samples

**Fig. 2:**
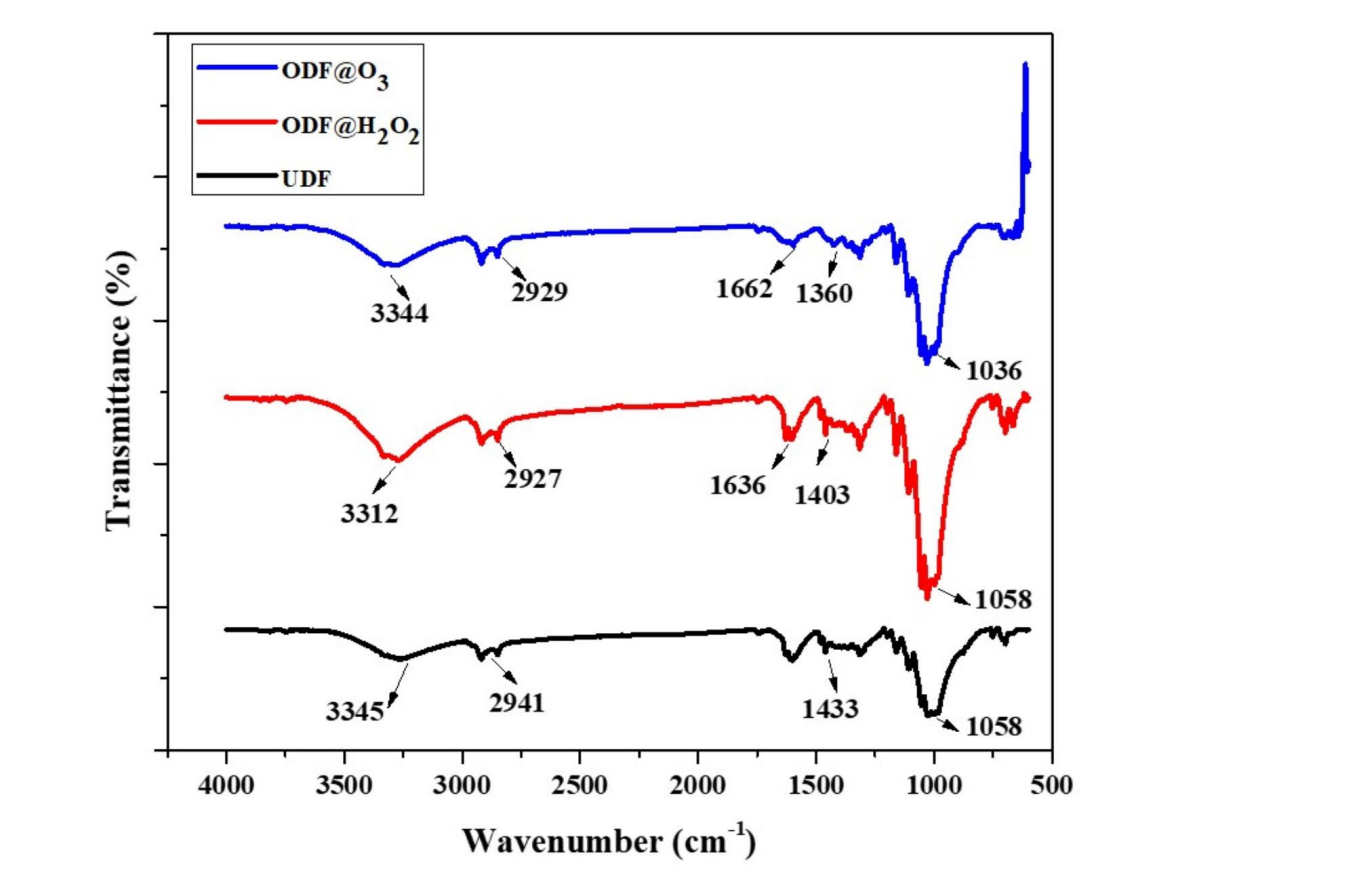
FTIR spectra of UDF, ODF@H_2_0_2_, and ODF@0_3_.

Once the samples with the highest capacity to absorb cadmium ions from water have been shortlisted, various analysis of those samples were conducted. Figure 2 shows the FTIR of untreated and treated denim fabrics. The FTIR spectrum of untreated denim fabric exhibits peaks that originated from cellulose macromolecule and indigo dye at 3345 cm^−1^ attributed to O–H stretching, 2941 cm^−1^ related to C–H aromatics, 1589 cm^−1^ for N–H stretching, 1433 cm^−1^ and 1058 cm^−1^was due to C–N and C-O stretching respectively^17^. From the FTIR spectrum of (ODF@0_3_) and (ODF@H_2_0_2_), it can be observed that the peak at 1058 cm^−1^ i.e. the β-glucosidic bond in the cellulose macromolecules do not show any major variation between the untreated and treated fabrics. Furthermore, it was noted that the band at 3345 cm^−1^ due to OH stretching experienced a minor shift in the position to 3312 cm^−1^ and 3344 cm^−1^after the treatment with ozone and hydrogen peroxide respectively. This slight modification results in a reduction of the hydrogen bonds between the cellulose macromolecules^18^.

**Fig. 3:**
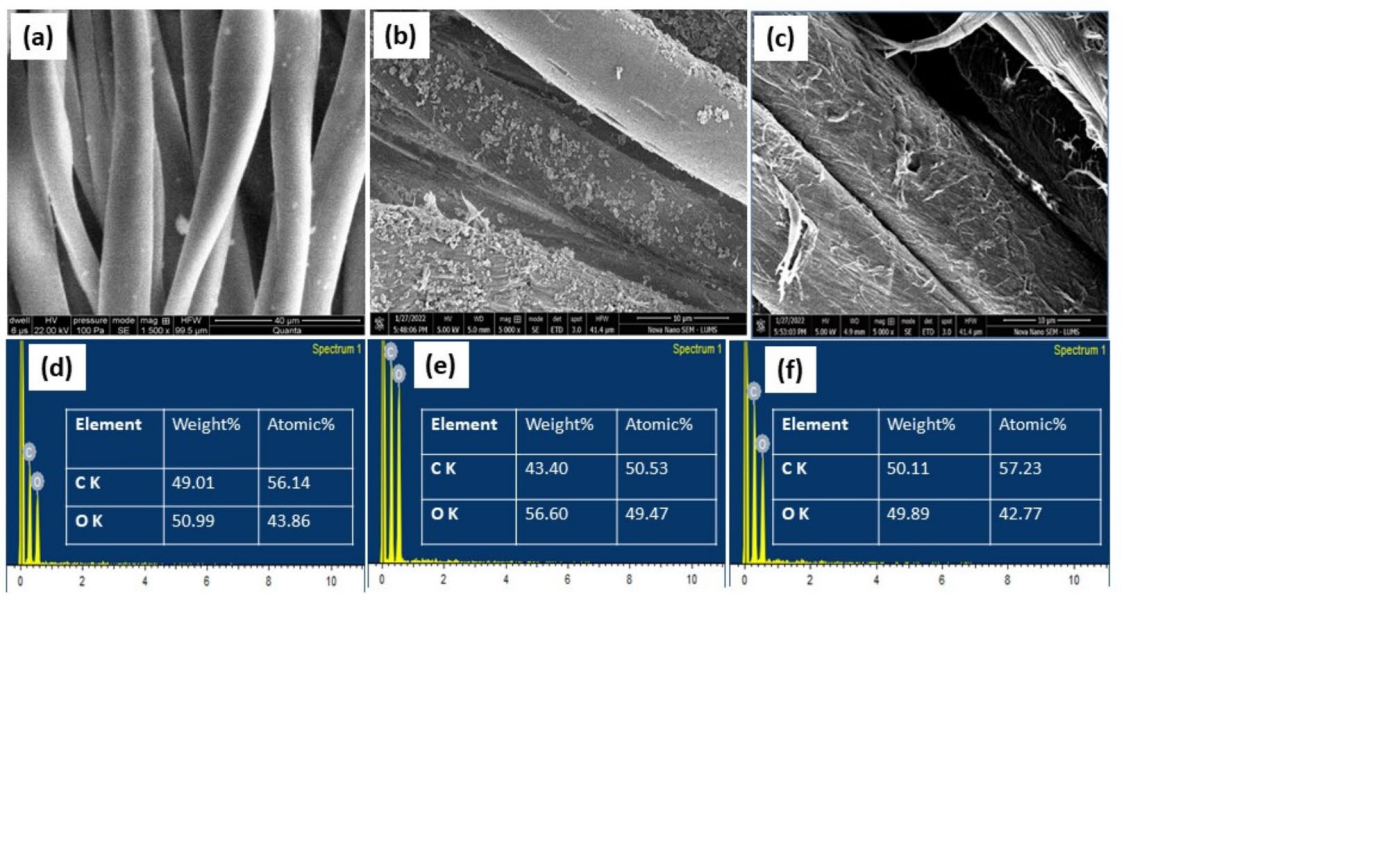
SEM images of UDF (**a**), ODF@H_2_0_2_ (**b**) ODF@0_3_ (**c**) and EDX analysis of UDF (**d**), ODF@H_2_0_2_ (**e**) ODF@0_3_ (**f**).

Figure 3a shows the scanning electron microscopic image of UDF. Figure 3b shows the fabric treated with ODF@03 whereas, Fig. 3c is that of ODF@H_2_0_2_. The ozone treatment made the surface of fibers very rough due to oxidation of cellulosic chains. The treatment with hydrogen peroxide happens to be very drastic. It causes breakage of cellulosic chains due to excessive oxidation which leads to generation of beads on fibers. EDX analysis of UDF, ODF@H_2_0_2_, and ODF@0_3_ was performed to analyze the different elements and their weight percentages. Figure 3d and e, and 3f show the results of treated and untreated denim fabrics. The weight% of oxygen elements enhanced in ODF@0_3_ due to the oxidation of denim fabric Fig. 3f while, ODF@H_2_0_2_ has less weight% of oxygen elements Fig. 3e relative to the UDF Fig. 3d, and ODF@0_3_ Fig. 3 f. which may be attributed to loss of oxygen due to formation of smaller volatile molecules like carbon dioxide. The exact values of weight% of different elements are represented in Fig. 3d and e, and f.

**Fig. 4:**
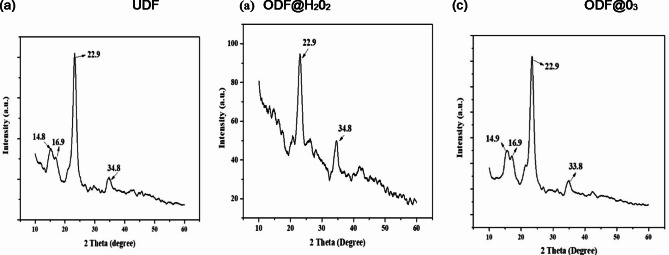
XRD analysis of UDF (**a**), ODF@H_2_0_2_ (**b**) and ODF@0_3_ (**c**).

The XRD spectrum of prepared sorbents are represented in Fig. 4. XRD pattern of UDF exhibits peaks at 14.9, 16.9 22.9, and 34.8˚ which correspond to (1–10), (110), (200) and (004) crystalline planes respectively (Fig. 4a) which are associated with the crystalline structure of cellulose^18^. The XRD pattern of ODF@H_2_0_2_ is presented in Fig. 4b which shows that the peak intensity of peaks at 14.9˚ and 16.9˚ is very low whereas, there is no significant change in case of ozone treatment (ODF@0_3)_ Fig. 4c. This might be due to the oxidation of cellulose fibers in the denim fabric by hydrogen peroxide due to high concentration causing slight disintegration of crystalline structure, while the ozone treatment was just a surface treatment. However, the peak at 34.8˚ remained unaffected during hydrogen peroxide treatment which corresponds to plane (004) and is characteristic peak of cellulose (II). This plane is formed during mercerization (caustic treatment) due to staking of crystallographic layers and has very strong hydrogen bonding which resists disintegration of structure due to hydrogen peroxide treatment in ODF@H_2_0_2_.

## Sorption experiment

### Sorption isotherm

The effect of different initial Cd concentrations (5, 25, 50, 100, 200 and 400 ppm) on the removal of Cd from water was tested using 0.01 g of both sorbents dose, at normal pH and optimum shaking time. The plot of equilibrium metal concentration against experimental sorption capacities is presented in supplementary Fig. 1a and 1b. It was noticed that by increasing the initial concentration, the sorption capacity rapidly increased. Figure shows the high sorption efficiency at low initial concentration by exhibiting a sharp slope, while exhibiting a flat slope at higher concentration which indicates saturation of sorption at higher concentration of metal. The increment in metal sorption potential may be due to diffusion-based mechanism on both sorbents^19^ and the higher metal concentration also increases the contact opportunities and provides a strong driving force to react with the functional groups present on the surface of sorbents ODF@H_2_0_2_ and ODF@0_3_. The maximum sorption capacity was achieved at 100 ppm metal concentration. After that a decrease in removal % was recorded. This decrease in sorption rate after 100 ppm metal concentration may be due to the insufficient binding sites pertaining to saturation at very high metal concentrations^20^. To further understand the sorption mechanism on ODF@H_2_0_2_ and ODF@0_3_, the obtained experimental sorption results were fitted with Freudlich, Langmuir and Temkin models. The Langmuir model assumes the monolayer sorption of adsorbate on the surface of adsorbent^21^. The Langmuir equation can be represented as

1 $$\:\frac{1}{{q}_{e}}=\frac{1}{{q}_{max}.{K}_{L}}\frac{1}{{C}_{e}}+\frac{1}{{q}_{max}}$$

where, q_e_ (mg g^−1^) represents the equilibrium sorption capacity of metal ions on surface, q_max_ (mg g^−1^) represents the maximum sorption capacity of metal ions at complete monolayer coverage. C_e_ (mg L^−1^) is the concentration of metal ions at equilibrium. K_L_ (L mg^−1^) is the Langmuir constant related to the heat of sorption^21^.

The Freundlich model describes the sorption behavior for sorbents having heterogenous surfaces and assumes multilayer sorption of adsorbate on the heterogenous surface of adsorbent. The Freundlich equation can be represented as:

2 $$\:\text{l}\text{n}{q}_{e}=\:\frac{1}{\text{n}}\text{l}\text{n}{C}_{e}+\text{l}\text{n}{K}_{f\:}$$

where, qe (mg g^−1^) represents the equilibrium sorption capacity of metal ions on surface. Ce (mg L^−1^) is the concentration of metal ions at equilibrium. K_f_ (mg g-1) is the Freundlich sorption isotherm constant related to sorption capacity. 1/n represents the magnitude of driving force for sorption and indicates the heterogeneity of the data distribution.

The Temkin equation can be represented as

3 $$\:{q}_{e}=\frac{\text{R}\text{T}}{{b}_{T}}\text{l}\text{n}{K}_{t}+\frac{\text{R}\text{T}}{{b}_{T}}\text{l}\text{n}{C}_{e}$$

where, qe (mg g^−1^) represents the equilibrium sorption capacity of metal ions on surface. K_t_ (L g^−1^) is the Temkin sorption isotherm constant. T is the absolute temperature (K). R represents the ideal gas constant 8.314 (J mol^−1^K). b_T_ is the constant related to the heat of sorption (Jmol^−1^).

**Fig. 5:**
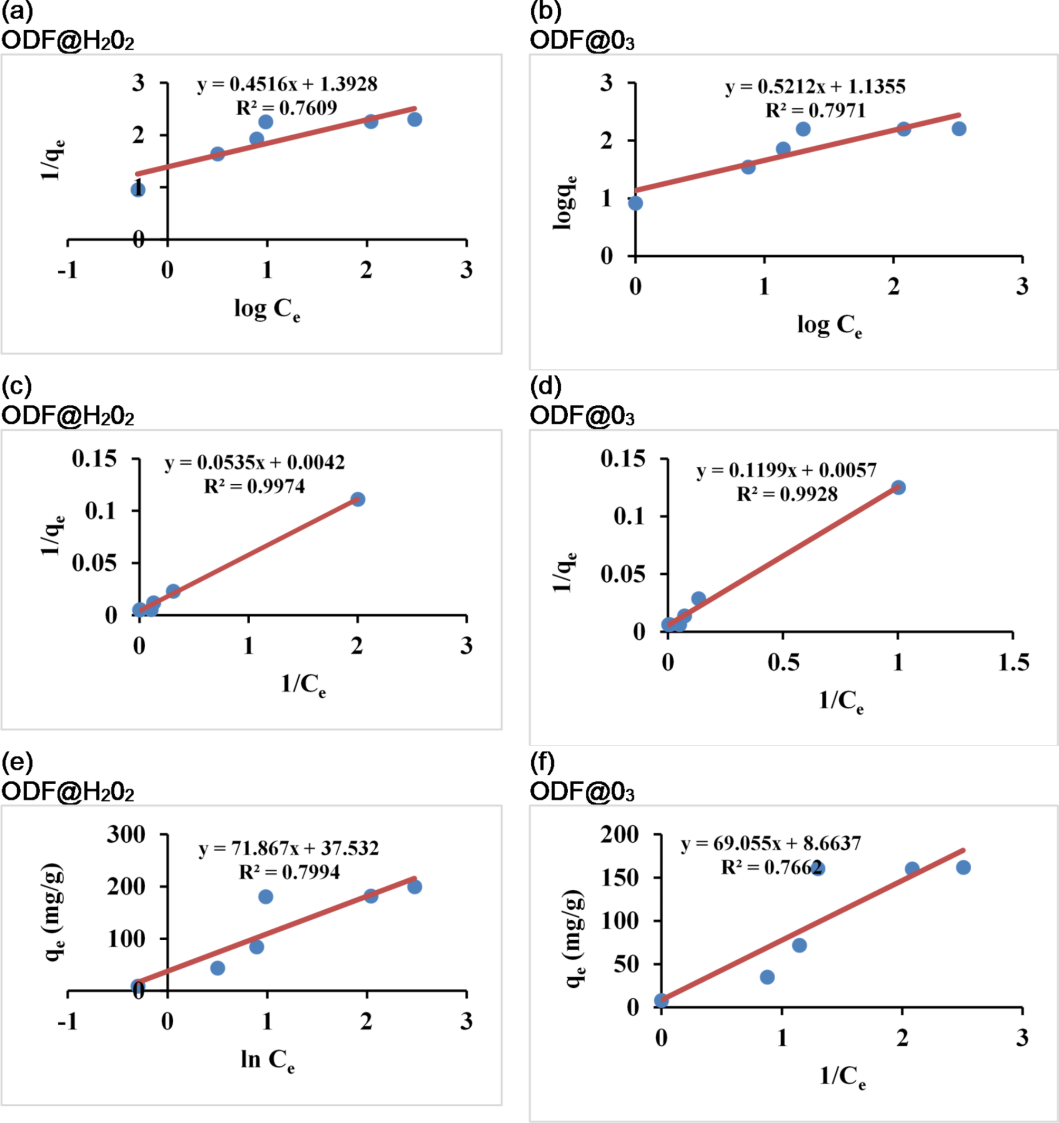
Sorption isotherms for Freundlich (a and b) Langmuir (c and d) and Temkin (e and f) for the sorption of Cd on ODF@H_2_0_2_ and ODF@0_3_ respectively.

The fitting results for three models have been presented in Fig. 5a and f. and the model’s calculated parameters have been presented in Table 1. It is clear from Fig. 5c and d, and Table 1 that Langmuir model best fitted with the sorption of Cd on ODF@H_2_0_2_ and ODF@0_3_ as compared to Freundlich (Fig. 5a and b) and Temkin model (Fig. 5e and f) as indicated by the higher correlation coefficients values (Table 1). This indicates that the sorption of Cd on ODF@H_2_0_2_ and ODF@0_3_is monolayer and homogenous^22–24^. The maximum sorption capacities for Cd calculated from Langmuir model were 238.1 and 175.4 mg g^−1^ for ODF@H_2_0_2_ and ODF@0_3_ respectively indicating that ODF@H202 has 36% more sorption potential for Cd compared to ODF@0_3_. The separation factor R_L_ was calculated for Langmuir model to estimate the extent of sorption using the following Eq. 

4 $$\:{R}_{L}=\frac{1}{1+{K}_{L}{C}_{o}}$$

When the value of RL is 0 the sorption is irreversible, beneficial when 0 < RL < 1 and adverse when RL > 1^25^. The RL values we achieved for both ODF@H_2_0_2_ and ODF@0_3_ were between 0 and 1, so the sorption of Cd on both sorbents is beneficial. Furthermore, both of our sorbents exhibited more sorption capacities compared to most of the previously reported sorbents. The comparison of maximum sorption capacities of ODF@H_2_0_2_ and ODF@0_3_ for Cd metal with other reported sorbents has been presented in supplementary Table 1.

**Table 1:** Parameters for Freundlich, Langmuir and Temkin models.

Adsorbent	Freundlich					Langmuir						Temkin				
	*n*	K_f_ (mg g^−1^)	*R* ^2^	RMSE	*P* value	Q_m_ (mg g^−1^)	b_L_	*R*_L_ (Lmg^−1^)	*R* ^2^	RMSE	*p* value	b_T_ (J mol^−1^)	K_T_ (L g^−1^)	*R* ^2^	RMSE	*p* value
ODF@H_2_0_2_	2.21	4.03	0.761	0.28	0.005	238.09	0.08	0.33	0.997	0.002	0.02	34.49	1.68	0.799	40.8	0.22
ODF@0_3_	1.92	3.11	0.797	0.26	0.005	175.44	0.05	0.50	0.993	0.004	0.04	35.89	1.13	0.766	37.8	0.78

## Sorption kinetics

**Fig. 6:**
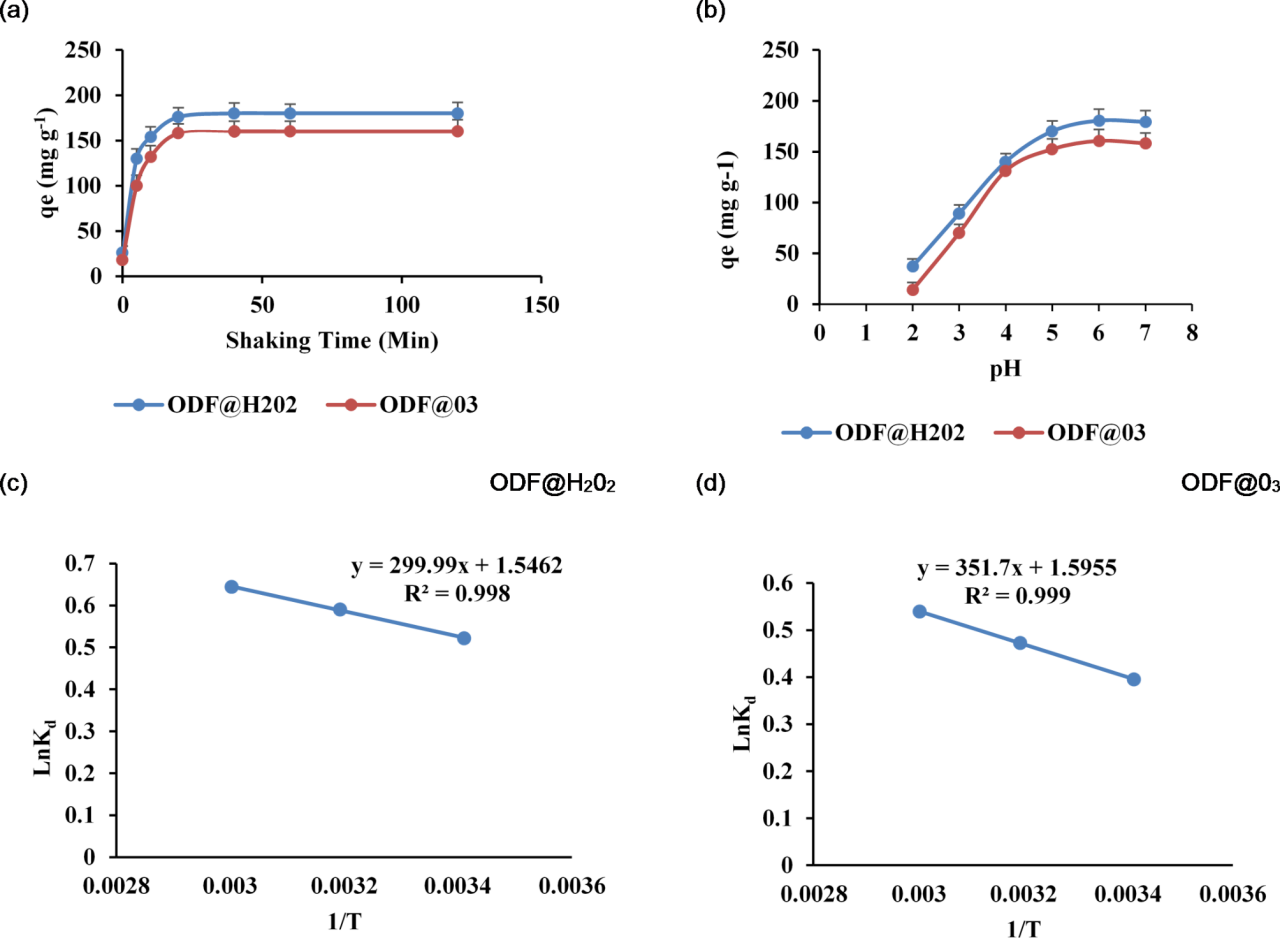
Effect of different shaking times (**a**) and effect of different pH (**b**) on the equilibrium sorption capacity of Cd by ODF@H_2_0_2_ and ODF@0_3_, The plot of (1/T) versus LnK_d_ for ODF@H_2_0_2_ (**c**) and ODF@0_3_ (**d**).

The effect of contact time on the sorption of Cd on ODF@H_2_0_2_ and ODF@0_3_ was investigated using different shaking times (0, 5, 10, 20, 40, 60 and 120 min). Figure 6a shows the sorption capacities (q_t_) achieved as a function of time at three different initial concentrations 5, 50, and 100 mg L^−1^). It is clear from the figure that the sorption capacities rapidly increased with increasing contact time reaching to maximum at 20 min of contact time, after that became constant for both ODF@H_2_0_2_ and ODF@0_3_by reaching at equilibrium time^22^. The very.

short equilibrium time (20 min) as we achieved in this study for heavy metal removal by both ODF@H_2_0_2_ and ODF@0_3_ is beneficial for practical application of these sorbents for wastewater treatment. To further explore the sorption mechanism the experimental results were modeled by Pseudo first and Pseudo second order kinetic models. The equations for both models are given as under

5 $$\:\text{ln}\left(\text{q}\text{e}-\text{q}\text{t}\right)=\text{ln}\left(\text{q}\text{e}\right)-{K}_{1}\text{t}$$

6 $$\:\frac{t}{{q}_{t}}=\frac{1}{{K}_{2}{q}_{e}^{2}}+\frac{1}{{q}_{e}t}$$

Where K_1_ (min^−1^) and K_2_ (g mg^−1^ min^−1^) represent the constants of reaction rate for Pseudo first and Pseudo second order models respectively. The plots between time (min) and ln(q_e_-q_t_) for pseudo first order and between time (min) and t/q_t_ for pseudo second order have been presented in (supplementary Fig. 2a and 2b (ODF@H_2_0_2_) and Fig. 2b and c (ODF@0_3_). The calculated parameters for these models are given in Table 2. It is clear from Table 2 that Pseudo second order gave best fit for the sorption of Cd on both ODF@H_2_0_2_ and ODF@0_3_, exhibiting correlation coefficients (R^2^) values 0.9997 and 0.9993 for ODF@H_2_0_2_ and ODF@0_3_respectively compared to Pseudo fort order R^2^(0.6278 and 0.5674) respectively^26;27^, Furthermore, the q_e_ values calculated from Pseudo second order (181.8 and 161.3 mg g^−1^) for ODF@H_2_0_2_ and ODF@0_3_ respectively at initial concentration 100 mg L^−1^ are very close to the experimental values i.e. (180.5 and 160.5 mg g^−1^) respectively indicating best fitting of Pseudo second order model for the Cd sorption. These findings can be supported by previous published work for Pb sorption^22^and Cd sorption^23^. So, the Cd sorption kinetics in our study followed pseudo second order kinetics and is governed by chemical interactions between metal ions and the abundance of COOH groups present (Fig. 2) on the surfaces of ODF@H_2_0_2_ and ODF@0_3_.

**Table 2:** Calculated parameters for Pseudo first and Pseudo second order models.

Adsorbent	Pseudo first order					Pseudo second order				
	**q**_**e**_ **(mg g** ^**−1**^ **)**	**k**_**1**_ **(min** ^**−1**^ **)**	**R** ^**2**^	**RMSE**	**p value**	**q** ^**2**^_**e**_ **(mg g** ^**−1**^ **)**	**k**_**2**_ **(g mg** ^**−1**^ **min** ^**−1**^ **)**	**R** ^**2**^	**RMSE**	**p value**
ODF@H_2_0_2_ ODF@0_3_	28.99 20.40	0.0003 0.0004	0.627 0.567	1.65 1.73	0.01 0.01	181.8 161.3	0.005 0.006	0.999 0.999	0.004 0.007	0.06 0.07

## Effect of pH

The solution pH is an important factor affecting the sorption of Cd by ODF@H_2_0_2_ and ODF@0_3_ sorbents. The surface electrical properties of ODF@H_2_0_2_ and ODF@0_3_ as well as the forms of Cd in the solution can vary with the change in solution pH. To investigate the effect of different solution pH on the sorption of Cd by ODF@H_2_0_2_ and ODF@03, we changed the pH from 2 to 7 while other batch conditions were kept constant as in previous sections and the results are shown in Fig. 6b. The minimum Cd sorption capacities were recorded at pH 2 i.e. 37 and 14 mg g^−1^ by ODF@H_2_0_2_ and ODF@0_3_respectively. As at low pH the surface of both sorbents is positively charged and Cd is least sorbed on these surfaces due to electrostatic repulsion^28^. A sharp increase in the sorption capacities was noted with increasing solution pH and maximum sorption capacities (180.5 and 160.5 mg kg^−1^) for both ODF@H_2_0_2_ and ODF@0_3_ were recorded at Ph 6. When we increase the pH the surface functional groups of both ODF@H_2_0_2_ and ODF@0_3_are deprotonated making their surfaces gradually negatively charged and at high pH due to electrostatic attraction the positively charged Cd ions are more sorbed on the surfaces of both sorbents. These findings can be supported by^23,26^.

## Sorption thermodynamics

To investigate the effect of different temperatures on the sorption of Cd ions from contaminated water by ODF@H_2_0_2_ and ODF@0_3_we conducted a thermodynamic study at three different temperatures i.e. 293.15, 313.15 and 333.15 K under optimized pH conditions. For both sorbents a significant increase in equilibrium sorption capacities was recorded with increasing temperature. The thermodynamic parameters like Entropy change, Enthalpy change, and Gibbs free energy can provide the better information regarding spontaneity and thermodynamic changes during sorption processes^29^. The equations being used are as under

7 $$\:{\varDelta\:G}^{o}=-RTln{K}_{d}\:$$

8 $$\:\varDelta\:G=\varDelta\:{\text{H}}^{o}-T{\varDelta\:S}^{o}$$

Where, ΔS^o^ is the entropy change, ΔH^o^ is the enthalpy change, ΔG^o^ represents the Gibbs free energy related to sorption process, K_d_ is the distribution coefficient calculated by dividing equilibrium sorption capacity (Q_e_) over initial Cd concentration (C^o^). The calculated thermodynamic parameters have been presented in Table 3.

**Table 3:** Thermodynamic parameters for the sorption of cd on ODF@H_2_0_2_ and ODF@0_3_.

Sorbents	Temperature (K)	∆G° (KJ mol^−1^)	∆H° (KJ mol^−1^)	∆S° (J mol^−1^ K^−1^)
ODF@H_2_0_2_	293.15	−1272.8605	2494.12	12.85
	313.15	−1529.8605		
	333.15	−1786.8605		
ODF@0_3_	293.15	−964.60075	2924.03	13.27
	313.15	−1229.90075		
	333.15	−1495.20075		

The plot of (1/T) versus LnK_d_ for both sorbents ODF@H_2_0_2_ and ODF@0_3_ have been shown in Fig. 6c and d. The ΔH^o^ and ΔS^o^ were calculated from the slope and intercept respectively Table 3. As we increased the temperature the distribution ratio K_d_ was also increased. A direct proportion between K_d_values and temperature indicates that the sorption process is endothermic^30^. We obtained negative values of Gibbs free energy (ΔG^o^) at all temperatures indicating the spontaneous sorption of Cd^2+^ ions onto both ODF@H_2_0_2_ and ODF@0_3_. Furthermore, the ΔG^o^ negative values decreased with increasing temperature which suggests that at high temperatures a stronger adsorptive force exists between sorbents and Cd^2+^metal cations. These findings can be supported by^22^. The positive values of entropy change (ΔS^o^) confirmed the structural changes in sorbents ODF@H_2_0_2_ and ODF@0_3_ and affinity of both sorbents for Cd^2+^metal cations^22,30^. The positive values of entropy change ΔS^o^ also confirmed the occurrence of ion replacement reaction and enhanced randomness at solid-solution interface during sorption of Cd^2+^ onto ODF@H_2_0_2_ and ODF@0_3_ Gubbuk, (2011) results for the sorption of Cu^2+^ ions onto sporopollenin surface supports our findings for Cd^2+^ ions sorption on ODF@H_2_0_2_ and ODF@0_3_^31^ .

### Regeneration study

**Fig. 7:**
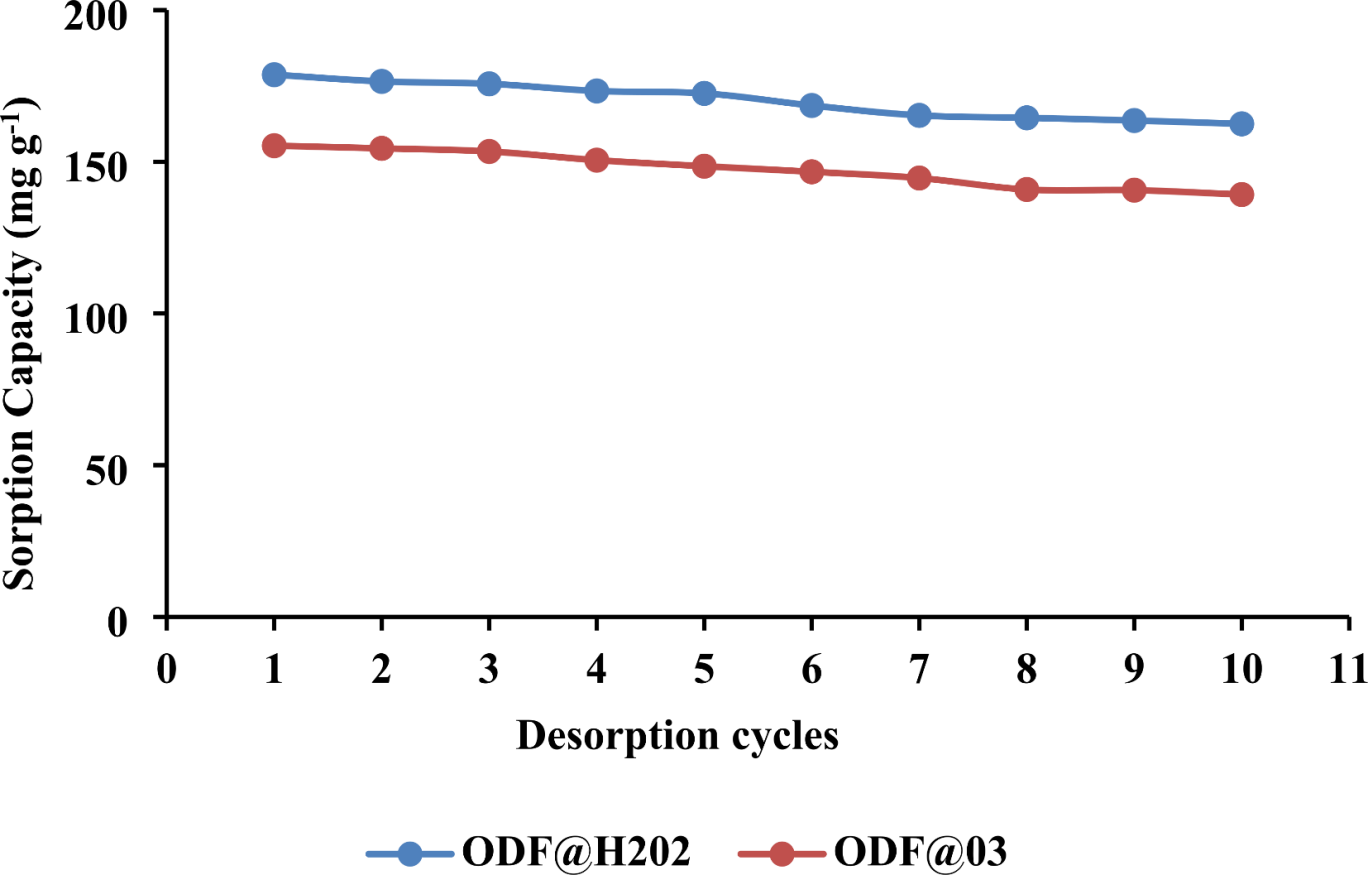
Desorption cycles of both sorbents.

A regeneration study was conducted to check the recycling ability of both ODF@H_2_0_2_ and ODF@0_3_ sorbents. The results of the regeneration study have been presented in Fig. 7. which shows that both sorbents exhibit minimal changes in the sorption efficiency during all 10 cycles. The sorbents ODF@H_2_0_2_ and ODF@0_3_ gave adsorption efficiency of 99 and 96% after 1 cycle of desorption. Whereas, after 10 recycles the ODF@H_2_0_2_ and ODF@0_3_ sorbents gave sorption efficiencies of 90 and 84% respectively. The efficient regeneration abilities of both sorbents indicate their suitability for their practical applications in wastewater treatment. Moreover, the cost of sorbent preparation from waste denim is nominal. The waste denim was collected from the local waste Collection Centre for free of cost. The treatment costs include hydrogen peroxide, sodium hydroxide and ozone treatment which is just a few cents per kilogram of sorbent.

## Conclusion

The present research work is designed to convert the challenge of landfill with waste denim into an opportunity to address the problem of heavy metal contamination of water. The waste denim fabric was oxidized with H_2_0_2_ and ozone under alkaline conditions using two simple techniques yielding ODF@H_2_0_2_ and ODF@0_3_. The spectroscopic and microscopic analysis confirmed the generation of new carboxylic groups on the surfaces and complete change in the surface morphology of both functionalized sorbents. The sorption efficiency of both sorbents was evaluated in a batch laboratory experiment. Both sorbents showed excellent sorption capacities for Cd from contaminated water. The ODF@H_2_0_2_ sorbent was found to be more effective by giving 36% more sorption capacity compared to ODF@03. The Langmuir and pseudo second order model best fitted with the experimental sorption results. The sorption isotherm, sorption kinetics and sorption thermodynamics revealed the sorption process as spontaneous and endothermic with chemisorption as a dominant process governing the sorption process. The present work addresses a practical approach as both sorbents can effectively be implied for wastewater treatment due to having efficient sorption capacities and recycling ability of 90% even after 10 recycles. Furthermore, our findings present a cost-effective and very simple technique where a waste is converted into a sustainable material having strong potential to address heavy metal contamination of water with minimum environmental impacts.

## Materials and methods

### Chemicals and materials

Hydrogen peroxide, sodium hydroxide (NaOH), sodium meta bisulfite and cadmium nitrate were procured from Merk Germany and were of analytical grade. The Cd stock and sub stock solutions were prepared in deionized water. The waste denim fabric was collected from disposed garments. All the glassware, centrifuge tubes, and laboratory consumables used in the experiment were dipped in nitric acid solution (20%) and then thoroughly washed three times with deionized water before use for quality assurance.

### Preparation of sorbent

The oxidation of denim fabric with hydrogen peroxide (H_2_O_2_) (named as ODF@H_2_0_2_) was done in the presence of a base in a beaker with stirrer and thermometer. A 10 cm^2^ piece of denim fabric was treated with different levels of H_2_O_2_ (5, 25, 50, 100, 200 and 400 ml/L) with 6 g of NaOH (sodium hydroxide) dissolved in distilled water. Continuous stirring was done on medium flame using Bunsen burner. When the temperature of the solution reached 85 ℃ the flame was slowed down, and further stirring was done for 30 min. After that, the fabric was removed and rinsed with distilled water. After washing it was placed on aluminum foil and dried in oven at 80 ℃ for 15 min.

**Fig. 8:**
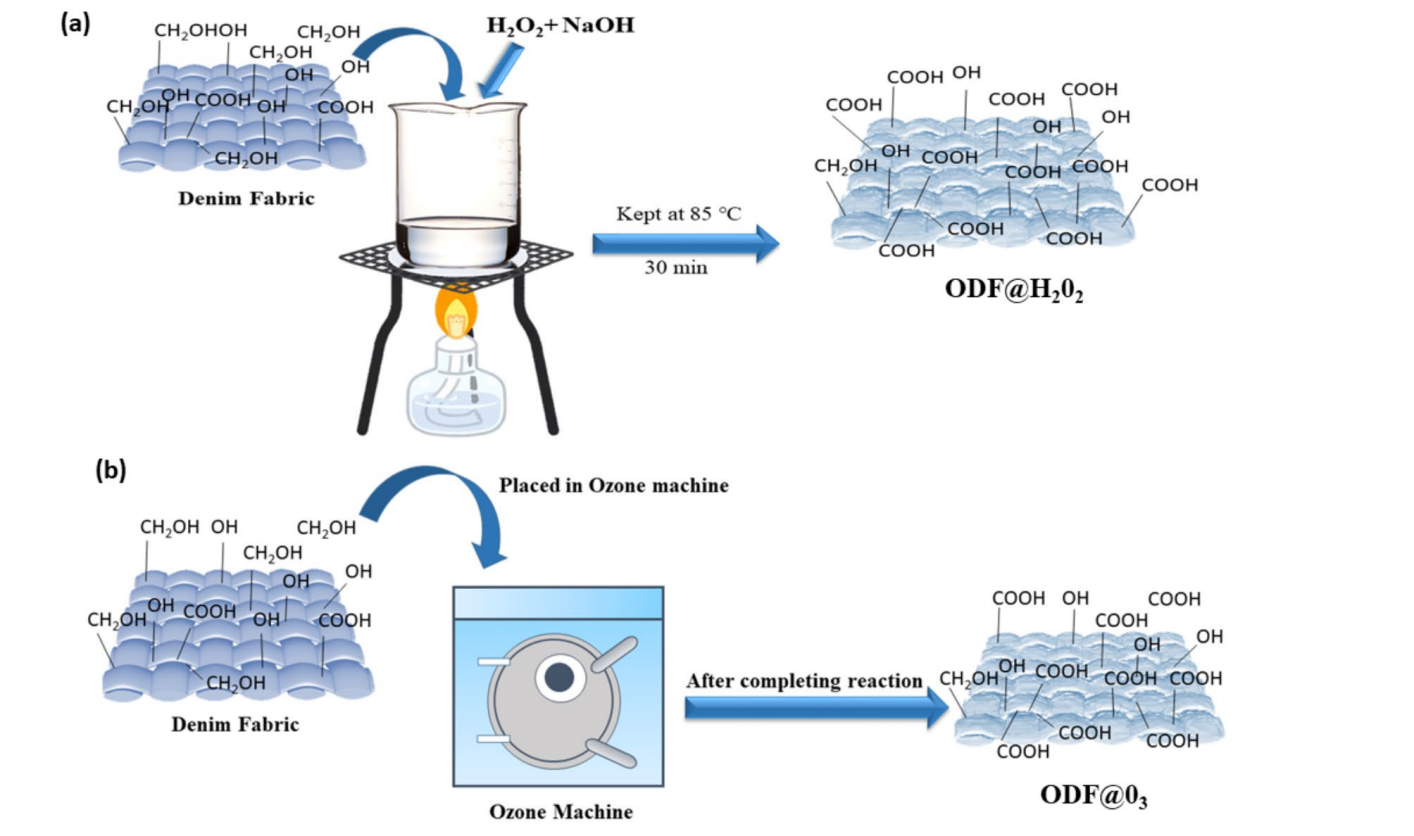
Schematic diagram for the preparation of sorbents.

A second piece of fabric was treated with ozone to generate carboxylic acid groups (named as ODF@0_3_). For this, an A4 size piece of moistened fabric was placed in the ozone treatment machine from Tonello (denim fading machine) that contains (0_3_ 48 g/h with 100% moisture) in air. Five samples of fabrics were run separately on ozonation machine with different spinning times (15, 30, 45, 60 and 75 min). To neutralize the fabric, sodium meta bisulfite (2 g/L) was used for 8 min. Adjusted the rotation at 27 rpm for 4 min. Then the first Drain was done by filling water 5 L/kg for 2 min. After the second drain, spinning was done at 200 rpm for 4 min, and then fabric was taken out from machine. The fabric was Squeezed and placed on aluminum foil and dried in the oven at 80 ℃ for 15 min. the schematics for the preparation of both sorbents has been presented in the Fig. 8a and b.

### Batch sorption experiment

A batch sorption experiment was conducted in the laboratory to optimize the different factors affecting Cd sorption on ODF@H_2_0_2_ and ODF@0_3_. A stock solution (1000 ppm) of Cd was prepared in deionized water using Cd (NO_3_)_2_·4H_2_O from which further sub stocks of 5, 25, 50, 100, 200, and 400 ppm concentrations were prepared. A sorbent sample of 0.01 g of both ODF@H_2_0_2_ and ODF@0_3_ was inserted in the 20 ml Cd solutions of different concentrations as stated above in 50 ml centrifuge tubes. The centrifuge tubes were shaken on an orbital shaker for 1 h at 180 rpm at 25 ℃. Then the sorbent material was removed from the solution and the solution mixture was filtered by filter paper. The atomic absorption spectrophotometer (AAS) was used to examine the remaining metal ion concentration after sorption. The sorption experiment was repeated 3 times and results were taken as an average of three replicates. The sorption trial was further performed using different shaking times of (0, 5, 10, 20, 40, 60, 120) min, different pH (2, 3, 4, 5, 6, and 7) different temperatures (293.15, 313.15 333.15) K while keeping the other batch conditions constant. The experimental sorption capacities (mg g^−1^) and metal removal % were calculated using the following Eq. 

9 $${\text{q}}_{\text{e}}={\left(\frac{{c}_{o-{C}_{e}}}{m}\right)}\text{V}$$

10 $$\:R=\left(\frac{{C}_{o}-{C}_{e}}{{C}_{o}}\right)100\:$$

Where C_o_ is the initial concentration and C_e_ is the equilibrium concentration of the cadmium, V is the volume in (L) and m is the quantity of sorbent in (g). The experimental results obtained allowed the determination of the sorption isotherm in the system Q = f(C_e_) and Cd removal curves in the system of R = f(Co).

### Regeneration study

To check the sustainability of the two prepared sorbents a series of desorption steps were conducted using the same treated fabrics for all cycles. The sorbents were treated with nitric acid to remove Cd ions from fabric. For this purpose, both sorbents removed from batch study from 100 ppm Cd solution were thoroughly washed with deionized water and placed in 15 ml HNO_3_ (1 mol/L) for 1 h. After that the sorbents were dried in oven and reused to check the sorption capacity in new 100 ppm Cd solution. The batch conditions were kept the same as mentioned above. This desorption process was repeated for 10 cycles using the same sorbents and sorption capacity was calculated for each cycle.

## Electronic supplementary material

Below is the link to the electronic supplementary material.

Supplementary Material 1

## Data Availability

The datasets used or analyzed during this research work are available from the corresponding author upon request.
