# Supplementary material for: Valorization of textile waste for removal of Cadmium from contaminated water
Source: Sci Rep. 2024 Dec 23;14:30611. doi: 10.1038/s41598-024-82456-x (PMC11666537; doi:10.1038/s41598-024-82456-x)
Supplement: Supplementary file 1 — Supplementary Material 1 [file 41598_2024_82456_MOESM1_ESM.docx]

**Valorization of textile waste for removal of Cadmium from contaminated water**

Humera Aziz^1*^, Munir Ashraf^2^, Muhammad Rizwan^1^, Umair Riaz^3^, Saba Akram^4^, Ali Raza^5^ and Jean Wan Hong Yong^6*^

1. Department of Environmental Sciences, Government College University, Faisalabad, 38040, Pakistan
2. School of Culture and Design, Clothing Technology, HTW Berlin –University of Applied Sciences for Technology and Economics Berlin, Germany
3. Department of Soil and Environmental Sciences, MNS-University of Agriculture, Multan, 60000, Pakistan
4. Functional Textiles Research Group, School of Engineering and Technology, National Textile University, Faisalabad-38000, Pakistan
5. Faculty of Veterinary Sciences, University of Veterinary and Animal Sciences, Lahore, 54000, Pakistan.
6. Department of Biosystems and Technology, Swedish University of Agricultural Sciences, 23456 Alnarp, Sweden

Correspondence*: [humeraaziz.uaf@gmail.com](mailto:humeraaziz.uaf@gmail.com); [jean.yong@slu.se](mailto:jean.yong@slu.se)

| 1. ODF@H_2_0_2_ | (b) ODF@0_3_ |
| --- | --- |

Fig. S1 experimental sorption isotherm of both sorbents

| (a) ODF@H_2_0_2_ | (b) ODF@0_3_ |
| --- | --- |
| (c) ODF@H_2_0_2_ | (d) ODF@0_3_ |

Fig. S2 plots of Pseudo first order model for ODF@H_2_0_2_ (a) and ODF@0_3_ (b); plots of pseudo second order model for ODF@H_2_0_2_ (c) and ODF@0_3_ (d)

| Metal ion | Sorbent | Sorption capacity (mg g^-1^) | References |
| --- | --- | --- | --- |
| Cd | PASP/CMPP | 56.8 | 1 |
| Cd | (Cd(II)-IIP) | 80.21 | 2 |
| Cd | SZF@CBC | 183.93 | 3 |
| Cd | GBC | 29 | 4 |
| Cd | MBC | 122.41 | 5 |
| Cd | Mag-SiO_2_-Si-AN. | 65 | 6 |
| Cd | AT-CP | 39.12 | 7 |
| Cd | Cel/PAN/AO | 123.23 | 8 |
| Cd | ZIF-8-EDA | 294.11 | 9 |
| Cd | RB700 | 119.33 | 10 |
| Cd | HA_mod_Fe | 76.2±2 | 11 |
| Cd | ODF@H_2_0_2_ | 238.09 | This study |
| Cd | ODF@0_3_ | 175.44 | This study |

Table S1 comparison of the sorption capacities of the sorbents of this study and previously published sorbents

References:

1. Zhang, F. et al. Preparation of hydrogels based on poplar cellulose and their removal efficiency of Cd (II) from aqueous solutions. *Journal of Water and Health*.  *21*(6), 676-686. (2023).
2. Ding, Z. et al. Controllable synthesis of 3D superhydrophilic Cd (II) ion-imprinted polymer microspheres based on OV-POSS and bifunctional monomers synergy with superior selectivity for Cd (II) adsorption. *Colloids and Surfaces A: Physicochemical and Engineering Aspects*. *676*, 132169. (2023).
3. Xia, H. et al. Unraveling adsorption characteristics and removal mechanism of novel Zn/Fe-bimetal-loaded and starch-coated corn cobs biochar for Pb (II) and Cd (II) in wastewater. *Journal of Molecular Liquids*. *391*, 123375. (2023).
4. Yu, Y. et al. Capacity and mechanisms of Pb (II) and Cd (II) sorption on five plant-based biochars. *Sustainability*. *15*(9), 7627. (2023).
5. Liang, X. et al. The simultaneous high-effective removal of As (III) and Cd by a modified biochar derived from rice straw. *Journal of Environmental Chemical Engineering*. *11*(3), 109874. (2023).
6. Younes, E.A. El-Sheikh, A.H. & Alsmadi, R.B. The use of new class α-amino nitrile modified magnetic adsorbents for removal of Cd (II) from aqueous medium: Sorbent modification vs. α-amino nitrile addition to the adsorption medium. *Emerging Contaminants*. *10*(1), 100261. (2024).
7. Abdelmonem, H.A. et al. Cellulose-embedded polyacrylonitrile/amidoxime for the removal of cadmium (II) from wastewater: Adsorption performance and proposed mechanism. *Colloids and Surfaces A: Physicochemical and Engineering Aspects*. *684*, 133081. (2024).
8. Khosravi, A. et al. Enhanced adsorption and removal of Cd (II) from aqueous solution by amino-functionalized ZIF-8. *Scientific Reports*. *14*(1), 10736. (2024).
9. Wang, Y. et al. The immobilization of cadmium by rape straw derived biochar in alkaline conditions: Sorption isotherm, molecular binding mechanism, and in-situ remediation of Cd-contaminated soil. *Environmental Pollution*, *351*, 123969. (2024)
10. Linkevich, E.V. & Prokopyuk, V.M. Adsorption of Cd (II) and Cr (VI) by a Complex Humic Sorbent: Kinetic and Redox Characteristics of the Process. *Russian Journal of Physical Chemistry A*. 1-9. (2024).
11. Nkosi, N. et al. Binary adsorption of toxic nickel (II) and cadmium (II) ions from aqueous solution by acid modified chilli peppers. *Energy Nexus*. 100313. (2024)
